# Effect of gastrointestinal digestion on the stability, antioxidant activity, and Caco‐2 cellular transport of pigmented grain polyphenols

**DOI:** 10.1111/1750-3841.17009

**Published:** 2024-03-11

**Authors:** Borkwei Ed Nignpense, Nidhish Francis, Christopher Blanchard, Abishek Bommannan Santhakumar

**Affiliations:** ^1^ School of Dentistry and Medical Sciences Charles Sturt University Wagga Wagga NSW Australia; ^2^ Gulbali Institute Charles Sturt University Wagga Wagga NSW Australia; ^3^ School of Agricultural, Environment and Veterinary Sciences Charles Sturt University Wagga Wagga NSW Australia

**Keywords:** Caco‐2 cellular transport, polyphenols, simulated digestion, pigmented cereal grains

## Abstract

Grain polyphenols are known to possess several health properties. However, their digestive stability and intestinal absorption have not been fully elucidated. This study investigated the fate of pigmented grain polyphenols in the digestive system. Purple rice, purple barley, purple wheat, and blue wheat extracts were subjected to simulated gastric and intestinal phase digestion, followed by Caco‐2 cellular transport. Phenolic profiling and antioxidant activity were determined using benchtop assays and an ultra‐high‐performance liquid chromatography–2,2′‐azino‐bis(3‐ethylbenzothiazoline‐6‐sulfonic acid free radical) (UHPLC‐ABTS^•+^) system. The results demonstrated a decrease in the total phenolic content of extracts after digestion, with purple rice extract retaining the highest phenolic content (79%) and ABTS^•+^antioxidant activity (31%). Antioxidant activity was retained the most during the gastric phase; however, dominant antioxidant compounds were not detected after intestinal digestion. Significant variations in phenolic composition and radical scavenging activity were detected after digestion. Protocatechuic acid, vanillic acid, apigenin, and chrysoeriol were all transported across the intestinal barrier. The findings of this study provide novel insights into the in vitro stability and antioxidant activity of cereal grain polyphenols after simulated digestion.

## 1 INTRODUCTION

Pigmented cereal grains are known to contain high concentrations of phenolic compounds, including phenolic acids, anthocyanins, flavanols, flavones, and other flavonoids (Ed Nignpense et al., 2022a). Several studies have shown that these compounds contribute to their color and bioactive health properties. For example, purple rice contains high anthocyanin content and its extracts have been shown to have strong radical scavenging activity and anti‐inflammatory and anti‐obesity effects ex vivo (Callcott et al., 2018). Black sorghum, which is rich in catechin and its derivatives, has strong free radical activity and cardioprotective effects ex vivo (Ed Nignpense et al., 2020; Francis et al., 2019). Pigmented wheat extracts, which are rich in anthocyanins and flavonoids, have demonstrated high antioxidant activities (Liu et al., 2010). Furthermore, pigmented barley, rich in flavan‐3‐ols, has shown potent antioxidant activity in vitro and in vivo (Shen et al., 2016). Phenolic‐rich extracts of pigmented varieties of rice, barley, and sorghum have been shown to induce anticarcinogenic effects (Rao et al., 2019).

Although several studies have demonstrated the bioactivity of raw extracts derived from cereal grains, phenolic compounds must be bioavailable to exert their potential health benefits in the human body. Bioavailability is defined as changes occurring in a particular drug or nutrient from ingestion to excretion (Manach et al., 2005). The bioavailability of polyphenols remains controversial, with some studies indicating that polyphenols may be poorly absorbed by the body owing to their complex molecular structure, interactions with other food components, and poor stability and solubility during gastrointestinal digestion (D'Archivio et al., 2010; Gamel et al., 2019; Manach et al., 2005). Nevertheless, knowledge about the bioavailability of grain polyphenols is critical for evaluating their potential health benefits and developing functional grain products that maximize these benefits.

Ideally, human dietary intervention clinical trials are the preferred model for investigating the bioavailability and bioactivity of grain polyphenols. However, due to the cost and ethical considerations associated with in vivo studies, in vitro digestion models in the recent past have effectively investigated bioaccessibility—the release of compounds during digestion (Ed Nignpense et al., 2021). This involves mimicking the physiological conditions of the human gastrointestinal tract during the oral, gastric, and intestinal phases. Recent studies have shown that rice, wheat, and barley compounds, including phenolic acids, anthocyanins, flavan‐3‐ols, and flavones, are progressively released during digestion with phenolic acids and flavones, showing greater bioaccessibility in the gastric phase than anthocyanins and flavan‐3‐ols (Drawbridge et al., 2021; Ed Nignpense et al., 2022a, 2022b). The previous studies examined in vitro digestion directly on whole grains rather than their phenolic extracts and thus did not focus on the changes in the phenolic composition during digestion. Rather, the results from the study showed that bioaccessible phenolics contained both free phenolics that survived digestion and phenolics released by digestion. However, conducting in vitro digestion directly on extracts will provide novel insights into the changes in phenolic composition and antioxidant properties during different phases of digestion. Coupled with an in vitro model of the intestinal epithelium, the human colorectal adenocarcinoma cell line (Caco‐2), the in vitro bioavailability of the digested phenolic compounds can also be investigated.

Therefore, this study aimed to investigate the fate of polyphenols derived from pigmented grain extracts following in vitro digestion using a Caco‐2 cell culture model. Phenolic extracts of purple rice (PRx), purple barley (PBx), purple wheat (PWx), and blue wheat (BWx) were used in this study. The objectives of this study were to evaluate the impact of in vitro digestion on the phenolic composition and antioxidant activity of pigmented grain extracts and to investigate the bioavailability of the digested polyphenols using Caco‐2 cellular transport.

## 2 MATERIALS AND METHODS

### 2.1 Materials

Chemicals, including methanol, hexane, sodium chloride, potassium chloride, sodium carbonate, sodium bicarbonate (NaHCO_3_), butylated hydroxytoluene, acetic acid, acetonitrile, and formic acid, were purchased from Chem Supply Ltd. (Port Adelaide, SA, Australia). All chemicals and solvents used were of analytical grade. Materials for digestion, including hydrochloric acid (HCl), bile extract, pepsin, and pancreatin enzymes, were obtained from Sigma‐Aldrich (St Louis, MO, USA). Reagents for benchtop assays, including 2,2′‐azino‐bis (3‐ethylbenzothiazoline‐6‐sulfonic acid) (ABTS), 2,4,6‐tris(2‐pyridyl)‐s‐triazine, 6‐hydroxy‐2,5,7,8‐tetramethylchroman‐2‐carboxylic acid (Trolox), and 2,4,6‐tri(2‐pyridyl)‐s‐triazine (TPTZ) were purchased from Sigma‐Aldrich (St. Louis, MO, USA). Phenolic standards, including protocatechuic acid, quercetin, rutin, gallic acid, ferulic acid, procyanidin B3, catechin, and luteolin, were sourced from Sigma‐Aldrich (St Louis, MO, USA). Materials for cell culture included Minimum Essential Medium Eagle (EMEM) (M4655), fetal bovine serum (FBS), and Transwell polycarbonate membrane cell culture inserts (Corning, CLS3412) purchased from Sigma‐Aldrich (St Louis, MO, USA). Penicillin–streptomycin and trypsin were obtained from Thermo Fisher Scientific (Waltham, MA, USA). Caco‐2 cells were purchased from the American Type Culture Collection (Manassas, VA, USA).

Four different pigmented cereal grains were used—purple rice (*Oryza sativa* L.), purple barley (*Hordeum vulgare* L.), purple wheat (*Triticum turgidum*), and blue wheat (*Triticum aestivum*). Purple rice grains, provided by the Department of Primary Industries, were grown in 2021 in Mackay, Queensland. Pigmented barley and wheat grain samples were obtained in the same year from field trials conducted in Narrabri, New South Wales by Australian Grain Technologies.

### 2.2 Extraction

Wholegrain samples were milled into flour (particle size of 0.5 mm) and defatted with hexane to remove lipophilic compounds using the extraction procedure described by Ed Nignpense et al. (2022a). After air‐drying overnight, 10 g of the defatted milled wholegrain flour was reconstituted in 100 mL methanol and stirred for 3 h at room temperature to extract phenolic compounds. The extraction was performed twice, and the extracts were concentrated to 1 g/mL using a vacuum evaporator (Rotavapor R‐210 BUCHI Labortechnik, Flawil, Switzerland). The extraction and chemical analyses were performed in triplicate.

### 2.3 In vitro digestion of extracts

The static in vitro digestion model described by Ed Nignpense et al. (2022a) was used to determine the composition and antioxidant activity of the polyphenols during both the gastric and intestinal digestion phases of digestion. Three milliliters of extract were homogenized in 10 mL of saline solution (140 mM NaCl, 5 mM KCl, and 150 µM BHT) and sonicated for 30 min at 25°C. The mixture was then acidified to pH 2 using 1 M HCl, followed by the addition of 0.5 mL of pepsin solution (0.2 g in 5 mL of 0.1 M HCl) and incubation in a shaking water bath for 1 h at 37°C to allow for gastric digestion. To mimic the conditions in the small intestine, the gastric digesta were alkalinized to pH 6.9 using 1 M NaHCO_3_. This was followed by the addition of pancreatic bile solution (0.45 g of bile extract and 0.075 g of pancreatin in 37.5 mL of 0.1 M NaHCO_3_) to allow for digestion while the mixture was incubated in a shaking water bath for 2 h at 37°C. Aliquots from gastric and intestinal digestions were centrifuged at 3082 × *g* for 10 min. The supernatants were retrieved, lyophilized, and reconstituted in methanol to obtain a concentration of 1 g/mL, which was used for further analysis. A blank (digestion reagent without extract) was used to correct any background interferences from enzymes and buffers.

### 2.4 Total phenolic content

The Folin–Ciocalteu method described by Rao et al. (2018) was used to determine the total phenolic content of undigested and digested cereal grain extracts. One hundred and twenty‐five microliters of extract, gallic acid standard, or blank (methanol) were mixed with 125 µL of Folin–Ciocalteu reagent and 500 mL of deionized water, followed by incubation in the dark for 6 min at room temperature. After incubation, 1.5 mL of 7% sodium carbonate and 1 mL of deionized water were added to the mixture, followed by incubating for 90 min at room temperature. The absorbance of samples was measured at 725 nm using a microplate reader. A gallic acid standard curve was used to quantify samples, and the results were expressed as milligrams of gallic acid equivalents (GAE) per 100 g dry‐weight flour.

### 2.5 Ferric reducing antioxidant power assay

The ferric reducing antioxidant power (FRAP) assay method described by Rao et al. (2018) was used to determine the antioxidant activity of samples. FRAP reagent was prepared by combining acetate buffer (300 mM, pH 3.6), TPTZ solution (10 mM TPTZ in 40 Mm HCl), and 20 mM FeCl_3_.6H_2_0 at a ratio of 10:1:1. Sixty microliters of extract, Trolox standard, or blank (methanol) were combined with 1.8 mL of FRAP reagent and 180 µL of deionized water. The mixture was gently vortexed and incubated for 40 min at 37°C. After incubation, the absorbance of the mixture was measured at 593 nm using a microplate reader. A Trolox standard curve was used to quantify samples and FRAP values were presented as milligrams of Trolox equivalents (TEs) per 100 g dry weight of cereal flour.

### 2.6 Total ABTS^•+^ radical scavenging activity assay

The method described by Saji et al. (2020) was used to evaluate the total ABTS^•+^ radical scavenging activity of the samples with some modifications. The ABTS stock solution was prepared by mixing 5 mL of 7 mM ABTS stock with 88 µL of 140 mM potassium persulfate and incubating the mixture in the dark for 12 h. The ABTS working solution was prepared by diluting the mixture with deionized water until the absorbance of 0.7 ± 0.05 at 735 nm was recorded. Thirty microliters of extract, Trolox standard, or blank (methanol) were combined with 3 mL of ABTS working solution and allowed to react for 10 min in the dark. The absorbance of the mixture was measured at 734 nm, and a Trolox standard curve was used to quantify samples.

The digested extracts were lyophilized and reconstituted to the same concentration as the undigested extract (1 g mL ^1^) before evaluating the total free radical scavenging activity using the following formula:

$$\begin{array}{ccc} & & \mathrm{Total}\;\mathrm{ABT}S^{•+}\;\mathrm{radical}\;\mathrm{scavenging}\;\mathrm{activity}\;\left(\%\right)\\ & & =\frac{\mathrm{Absorbance}\;\mathrm{of}\;\mathrm{control}\;-\;\mathrm{Absorbance}\;\mathrm{of}\;\mathrm{extract}}{\mathrm{Absorbance}\;\mathrm{of}\;\mathrm{control}}\;\times \;100. \end{array}$$

### 2.7 UHPLC‐online ABTS^•+^ analysis and identification of phenolic compounds

Phenolic characterization and antioxidant activity of extracts and their digesta were determined by chromatographic separation using an Agilent 1290 Infinity UHPLC system with a C18 Poroshell 120 column (3.0 × 100 mm, 2.7‐µm particle size) coupled with an online ABTS system, as described by Ed Nignpense et al. (2022b). A volume of 5.7 µL sample was injected into the system. The mobile phase consisted of 0.1% formic acid in deionized water (A) and 0.1% in acetonitrile (B) at a flow rate of 0.48 mL/min with the following elution gradient: 0−6 min, 0%−5.5% B; 6−32 min, 5.5%−30% B; 32−36 min, 30%−100% B.

The ABTS solution was injected at a flow rate of 0.32 mL/min with the aid of a coil column and binary pump. Antioxidant activity was detected using the online ABTS assay at 414 nm using a UV–vis detector. A diode array detector was used to identify and quantify anthocyanins and other polyphenols at wavelengths of 280 and 520 nm, respectively.

An Agilent 6530 Accurate‐Mass QTOF LC/MS was used to determine the mass spectra of the unknown UHPLC peaks. Samples were kept in an autosampler (4°C) to maintain stability. Mass detection was carried out using electrospray ionization with a gas nebulizer set at 45 psi, a drying nitrogen gas flow of 5 mL/min at 300°C, and a sheath gas flow of 11 L/min at 250°C. The capillary voltage was set to 3500 V and the nozzle voltage was set to 500 V. A full mass scan from 50 to 1300 *m*/*z* was used at a rate of two spectra per second in the negative ion mode. UHPLC peaks were analyzed using the Agilent Mass Hunter Qualitative Analysis software, as described by Ed Nignpense et al. (2022b). In the absence of pure standards, databases such as Metlin, ChemSpider, PubChem, and MassBank were used to provide tentative compound identification.

### 2.8 Caco‐2 cellular transport of polyphenols (intestinal absorption)

The Caco‐2 cellular transport experiment was performed according to the method described by Hilary et al. (2020), with some modifications. Caco‐2 cells (density 0.3 × 10^6^ cells/insert) were seeded on Transwell semi‐permeable membrane inserts (6‐well, 4.67 cm^2^, 0.4 µm pore size) and cultured in EMEM substituted with 15% FBS and 1% penicillin–streptomycin solution for 15 days at 37°C in a 5% CO_2_ incubator. The basolateral chamber received a medium devoid of cells. The cell culture medium was replaced every 3 days until the cells formed a monolayer. The formation and integrity of the monolayer were evaluated by measuring the transepithelial electrical resistance (TEER) using a MilliCell voltammeter (Millicell ERS‐2, Merck Millipore, Billerica, MA, USA). A TEER value exceeding 400 Ω/cm^2^ confirmed the formation and integrity of the monolayer. The digested grain extracts were diluted with media (1:1 vol/vol), injected into the Caco‐2 apical side of the monolayer, and incubated for 4 h at 37°C in a 5% CO_2_ incubator to mimic human gastrointestinal digestion conditions. At the end of the 4‐h incubation, apical and basolateral culture media were collected and freeze‐dried. Lyophilized samples were reconstituted with 1 mL of ultrapure water, filtered using a 0.22‐µm syringe filter, and the phenolic profile was analyzed as per Section 2.7.

### 2.9 Statistical analysis

Experimental data are presented as the mean ± standard deviation. Experiments were conducted in triplicate and analyzed using GraphPad Prism 8 software. One‐way analysis of variance followed by Tukey's post hoc multiple comparisons test was used to detect significant differences between groups. Statistical significance was set at *p* < 0.05.

## 3 RESULTS

### 3.1 Impact of simulated gastrointestinal digestion on the total phenolic content of pigmented grain extracts

Prior to simulated digestion, PRx had the greatest phenolic content (245 ± 22 mg GAE/100 g dw), followed by PBx (108 ± 45 mg GAE/100 g dw), BWx (46 ± 3 GAE/100 g dw), and PWx (34 ± 3 mg GAE/100 g dw). The extracts underwent significant changes in the total phenolic content after digestion. After the digestion of PRx, approximately 86% and 79% of its phenolic content were retained in the gastric and intestinal phases, respectively. There was a significant decrease (*p* < 0.001) in phenolic content between the undigested and intestinal phases of PRx. After digestion with PBx, approximately 59% and 55% of its phenolic content were retained in the gastric and intestinal phases, respectively, with a significant decrease (*p* < 0.001) detected after gastric digestion. BWx had no significant difference (*p* > 0.1) in the phenolic content before and after digestion, with approximately 63% and 93% of its phenolic content retained in the gastric and intestinal phases, respectively. After digestion of PWx, there was a significant increase (*p* < 0.01) in phenolic content, with about 114% and 126% of the phenolic content detected in the gastric and intestinal phases, respectively.

### 3.2 Impact of simulated gastrointestinal digestion on the ferric reducing antioxidant power of pigmented grain extracts

The total antioxidant activity of each extract, as determined by FRAP values, was significantly affected by digestion. Before digestion, PRx had the highest FRAP value (199 ± 12 mg TE/100 g dw), followed by PBx (127 ± 2 mg TE/100 g dw), PWx (36 ± 7 mg TE/100 g dw), and BWx (32 ± 3 mg TE/100 g dw). Notably, PRx retained about 91% and 17% of its antioxidant activity after gastric and intestinal digestion, respectively. In contrast, PBx retained about 35% and 9% of its antioxidant activity after gastric and intestinal digestion, respectively. PWx also experienced a significant decrease in antioxidant activity after intestinal digestion, with 47% of the activity being retained. BWx showed a significant decrease in antioxidant activity after digestion, but there was no difference in activity between the digestive phases.

### 3.3 Impact of simulated gastrointestinal digestion on the total ABTS^•+^ radical scavenging activity of pigmented grain extracts

The total ABTS^•+^ free radical scavenging activity was significantly affected by digestion. PRx had the greatest scavenging activity (89 ± 1%) in the undigested phase, followed by PBx (62 ± 11%), BWx (55 ± 4%), and PWx (33 ± 2%). After gastric digestion, the order of the extracts by decreasing antioxidant activity was PRx, PBx, PWx, and BWx. After intestinal digestion, the order was changed to PBx, PRx, BWx, and PWx. Notably, PRx retained the same scavenging activity after gastric digestion but significantly decreased to 31 ± 0% after intestinal digestion. PBx decreased from 71 ± 1% to 38 ± 3% scavenging activity after gastric and intestinal digestion, respectively. PWx also decreased significantly from 25 ± 1% to 12 ± 0% scavenging activity after gastric and intestinal digestion, respectively. BWx scavenging activity decreased from 23 ± 1% to 15 ± 0% after gastric and intestinal digestion, respectively.

### 3.4 Phenolic composition and antioxidant profile of extracts before and after digestion

Table 1 presents a list of 43 peaks representing the compounds detected in the ultra‐high‐performance liquid chromatography quadrupole time‐of‐flight mass spectrometry (UHPLC‐QTOF‐MS) profiles of both undigested and digested extracts. The phenolic composition of the extracts included two phenolic acids (P1 and P14), a phenolic amino acid tryptophan (P7), three anthocyanins (P13, P16, and P18), four flavan‐3‐ols (P3, P8, P10, and P12), six flavonols and their glycosides (P22, P24, P31, P34, P35, and P40), 12 flavones and their glycosides (P17, P20, P21, P27, P28, P29, P30, P32, P33, P36, P41, and P43), and other flavonoids (P23 and P25). All phenolic compounds were identified by matching the chromatographic data with analytical standards, online databases, and published literature. The unidentified peaks included unknown peaks identified before digestion (P2, P4, P11, P15, P19, P26, P39, P42) and those identified after digestion (P5, P6, P37, P38).

**TABLE 1:** Tentative identification, retention time (RT), and mass‐to‐charge ratio (*m*/*z*) of major chromatographic peaks detected in pigmented grain extracts before and after simulated digestion.

Peak	RT	*m*/*z*	Compound	Class
P1	5.6	153.0191	Protocatechuic acid	Phenolic acid
P2	6.3	671.2041	Unknown	
P3	6.7	305.0650	Gallocatechin	Flavan‐3‐ol
P4	6.8	655.2089	Unknown	
P5*	6.8	236.0553	Unknown digestive product	
P6◦	6.9	219.0507	Unknown digestive product	
P7	8.3	203.0829	Tryptophan	
P8	8.9	593.1299	Prodelphinidin B3	Flavan‐3‐ol
P9	10.5	559.2714	Unknown digestive product	
P10	11.2	289.0707	Catechin	Flavan‐3‐ol
P11	11.4	493.1560	Unknown	
P12	11.7	577.1357	Procyanidin B3	Flavan‐3‐ol
P13	13.2	447.0927	Cyanidin‐3‐glucoside (C3G)	Anthocyanin
P14^+^	14.7	167.0342	Vanillic acid	Phenolic acid
P15	14.7	787.3634	Unknown	
P16	16.7	461.1075	Peonidin‐3‐glucoside (P3G)	Anthocyanin
P17	17	563.1422	Apigenin 6‐C‐arabinoside‐8‐C‐hexoside	Flavone glycoside
P18	17.0	489.1039	Unknown anthocyanin	Anthocyanin
P19	17.5	387.0929	Unknown	
P20	17.6	563.1422	Apigenin‐6‐C‐arabinoside‐8‐C‐hexoside isomer 1	Flavone glycoside
P21	17.9	563.1422	Apigenin‐6‐C‐arabinoside‐8‐C‐hexoside isomer 2	Flavone glycoside
P22	18.5	479.0872	Myricetin‐7‐O‐glucoside	Flavonol glycoside
P23	19.1	303.0512	Taxifolin	Flavanonol
P24	20.2	463.0872	Isoquercetin	Flavonol glycoside
P25	20.4	461.1085	Tectoridin	Isoflavone
P26	21.4	475.1226	Unknown	
P27	21.5	461.1100	Chrysoeriol‐7‐O‐glucoside	Flavone glycoside
P28	22.4	769.1973	Apigenin‐8‐C‐sinapoylpentoside‐6‐C‐hexoside	Flavone glycoside
P29	22.8	769.1973	Apigenin‐8‐C‐sinapoylpentoside‐6‐C‐hexoside isomer	Flavone glycoside
P30	23.2	739.1866	Chrysoeriol 4′‐O‐pentoside‐7′ O‐rutinoside	Flavone glycoside
P31	23.5	317.066	Myricetin	Flavonol
P32	24.4	461.1077	Chrysoeriol‐7‐O‐glucoside isomer 1	Flavone glycoside
P33	24.7	475.0893	Chrysoeriol‐7‐O‐glucuronide	Flavone glycoside
P34	24.0	723.2128	Kaempferol 3‐rhamnosyl‐(1 → 4)‐rhamnoside‐7‐rhamnoside	Flavonol glycoside
P35	24.4	609.1811	Rutin	Flavonol glycoside
P36	24.5	461.1077	Chrysoeriol‐7‐O‐glucoside isomer 2	Flavone glycoside
P37*	25.6	177.0548	Unknown digestive product	
P38*	27.2	530.7950	Unknown digestive product	
P39	27.3	603.0766	Unknown	
P40	27.9	301.0353	Quercetin	Flavonol
P41	27.9	285.0000	Luteolin	Flavone
P42◦	29.37	489.1042	Unknown	
P43	32.17	299.0561	Chrysoeriol	Flavone

The symbol * indicates the compound was detected only in the intestinal phase. The **+** symbol indicates that the compound was detected after digestion in both the gastric and intestinal phases. The **◦** symbol indicates the compound was detected only in the gastric phase.

### 3.5 Changes to the phenolic composition before and after simulated digestion (gastric and intestinal phases) of pigmented cereal extracts

Digestion significantly affected the phenolic composition of purple rice extract (PRx), with 14 out of 16 compounds detected in the undigested phase recovered in the gastric phase and only 10 in the intestinal phase (Table 2). Compounds, including vanillic acid (P14), P5, P9, and P38, emerged after digestion. Anthocyanins, cyandi (P13) and P3G (P16), were the most abundant compounds in undigested PRx, but their amount significantly reduced after the gastric phase digestion to 13.84 ± 0.106 and 5.61 ± 0.227 mg GAE/100 g dw, respectively. Within the intestinal phase, the unknown compound, P38, was the most abundant at 1.204 ± 0.123 mg GAE/100 g dw. Purple barley extract (PBx) had 16 compounds, and only nine were recovered in the gastric phase, while five were recovered in the intestinal phase. The most abundant compound in undigested PBx was chrysoeriol 7‐O glucuronide (P33) at 3.178 ± 0.057 mg GAE/100 g dw, but after digestion, new compounds emerged as more abundant. P42 was the most abundant at 2.10 ± 0.096 mg GAE/100 g dw after the gastric phase and chrysoeriol (P43) was the most abundant at 1.17 ± 0.027 mg GAE/100 g dw after intestinal digestion. Purple wheat extract (PWx) had 12 compounds, and digestion resulted in a total of eight and nine compounds recovered in the gastric phase and intestinal phase, respectively. The compound P6 emerged after only gastric digestion. Tryptophan (P7) was the most abundant compound in both undigested and intestinal phases. Following gastric digestion, P2 and protocatechuic acid were the most abundant phenolic compounds at 0.919 ± 0.051 and 0.897 ± 0.026 mg GAE/100 g dw, respectively. Blue wheat extract (BWx) had 11 compounds, and digestion resulted in a total of five and six compounds being recovered in the gastric phase and intestinal phase, respectively. Tryptophan (P7) was the most abundant compound in both its undigested and intestinal phases, while apigenin 6‐C‐arabinoside‐8‐C‐hexoside (P17) and apigenin 6‐C‐arabinoside‐8‐C‐hexoside isomer (P21) were the most abundant compounds in the gastric phase at 0.518 ± 0.040 mg GAE/100 g dw and 0.481 ± 0.051 mg GAE/100 g dw, respectively. Overall, phenolic compounds showed decreased phenolic content after digestion of the extracts. Many compounds were reduced in amounts just after gastric digestion and others showed either trace or nil amounts. Compounds such as chrysoeriol‐7‐O‐glucuronide (P33) and chrysoeriol (P43) retained the same amount after digestion of PWx and BWx, respectively. Only one compound, tryptophan (P7), showed an increase in the amount after digestion.

**TABLE 2:** Changes to the phenolic composition before and after simulated digestion (gastric and intestinal phases) of pigmented cereal extracts.

		PRx mg GAE/100 g dw			PBx mg GAE/100 g dw			PWx mg GAE/100 g dw			BWx mg GAE/100 g dw		
Peak	Polyphenol class	Undigested	Gastric	Intestinal	Undigested	Gastric	Intestinal	Undigested	Gastric	Intestinal	Undigested	Gastric	Intestinal
P1	Phenolic acid	1.160 ± 0.058b	1.798 ± 0.128a	0.061 ± 0.009c	0.175 ± 0.008b	0.266 ± 0.019a		0.144 ± 0.006b	0.897 ± 0.026a	0.098 ± 0.003c	0.097 ± 0.013c	0.394 ± 0.033a	0.059 ± 0.004b
P2					1.257 ± 0.018a	0.315 ± 0.069b	0.296 ± 0.034b	0.689 ± 0.027b	0.919 ± 0.051a	0.288 ± 0.015c	1.231 ± 0.011c	0.370 ± 0.025a	0.482 ± 0.013b
P3	Flavan‐3‐ol				0.041 ± 0.009								
P4								0.078 ± 0.017			0.102 ± 0.010		
P5				0.641 ± 0.006									
P6									0.262 ± 0.012				
P7		0.341 ± 0.007b	0.300 ± 0.016c	0.915 ± 0.024a	0.356 ± 0.009b	0.275 ± 0.051c	1.423 ± 0.026a	4.102 ± 0.068b	0.260 ± 0.031c	6.241 ± 0.169a	5.979 ± 0.019a	0.356 ± 0.016c	5.129 ± 0.069b
P8	Flavan‐3‐ol				1.002 ± 0.031								
P9				1.164 ± 0.010									
P10	Flavan‐3‐ol				0.473 ± 0.005								
P11		0.507 ± 0.045a	Trace	Trace									
P12	Flavan‐3‐ol				0.943 ± 0.036								
P13	Anthocyanin	18.66 ± 0.803a	13.84 ± 0.106b		0.952 ± 0.013a	0.538 ± 0.055b		Trace	Trace		Trace	Trace	Trace
P14	Phenolic acid		0.58 ± 0.015a	0.134 ± 0.015b									
P15					1.840 ± 0.024a	0.665 ± 0.042b	1.148 ± 0.079a						
P16	Anthocyanin	9.43 ± 0.278a	5.61 ± 0.227b										
P17	Flavone glycoside							0.506 ± 0.10a	0.480 ± 0.030b	0.451 ± 0.006b	0.707 ± 0.019a	0.518 ± 0.040b	0.332 ± 0.016c
P18	Anthocyanin				1.975 ± 0.065a	0.425 ± 0.037b							
P19		0.243 ± 0.026											
P20	Flavone glycoside							0.166 ± 0.005b	0.225 ± 0.008a	0.077 ± 0.008c	0.236 ± 0.009a	Trace	0.092 ± 0.006b
P21	Flavone glycoside							0.777 ± 0.009a	0.645 ± 0.044b	0.360 ± 0.004c	1.066 ± 0.028a	0.481 ± 0.051b	0.345 ± 0.009c
P22	Flavonol glycoside	0.212 ± 0.019	Trace										
P23	Flavanonol	1.727 ± 0.226a	0.785 ± 0.099b										
P24	Flavonol glycoside	0.261 ± 0.037a	0.076 ± 0.013b	trace									
P25	Isoflavone	0.934 ± 0.157a	0.621 ± 0.005c	0.689 ± 0.040b									
P26		0.278 ± 0.028a	0.253 ± 0.016a	0.096 ± 0.005b									
P27	Flavone glycoside				0.358 ± 0.023	Trace							
P28	Flavone glycoside							0.219 ± 0.007a	0.193 ± 0.014b	0.105 ± 0.027c	0.111 ± 0.004	Trace	Trace
P29	Flavone glycoside							0.353 ± 0.01a	0.272 ± 0.023b	0.067 ± 0.004c	0.204 ± 0.014	Trace	Trace
P30	Flavone glycoside										0.080 ± 0.001	Trace	Trace
P31	Flavonol	0.323 ± 0.005a	0.253 ± 0.020b	0.044 ± 0.009c									
P32	Flavone glycoside				0.172 ± 0.002	Trace	Trace						
P33	Flavone glycoside				3.178 ± 0.057a	0.295 ± 0.013b	0.329 ± 0.017a						
P34	Flavanol glycoside	0.243 ± 0.011	Trace										
P35	Flavonol glycoside	0.816 ± 0.014a	0.410 ± 0.035b	0.049 ± 0.008c									
P36	Flavone glycoside				0.057 ± 0.005	Trace	Trace						
P37													0.160 ± 0.012
P38				1.204 ± 0.123			0.605 ± 0.042			0.615 ± 0.038			0.513 ± 0.064
P39		0.429 ± 0.018											
P40	Flavonol	0.456 ± 0.017a	0.282 ± 0.037b										
P41	Flavone				0.383 ± 0.006a	0.254 ± 0.02b	Trace	Trace	Trace	Trace			
P42						2.107 ± 0.096							
P43	Flavone				1.938 ± 0.020a	0.516 ± 0.023c	1.17 ± 0.027b	0.081 ± 0.003a	Trace	0.083 ± 0.001a			

Abbreviations: GAE, gallic acid equivalents; BWx, blue wheat extract; PRx, purple rice extract; PBx, purple barley extract; PWx, purple wheat extract, PWx. Different alphabets in a row indicate a significant difference in the amount of a compound before and after digestion of an extract.

### 3.6 Effect of digestion on online ABTS^•+^ antioxidant activity of phenolic compounds in grain extracts

The online ABTS^•+^ antioxidant activity profiles of all pigmented grain extracts were significantly altered by digestion (Table 3). Among the six antioxidant peaks detected in the undigested phase of PRx, only four were found in the gastric phase, and none were detected in the intestinal phase. The compounds cyanid (P13) and P3G (P16) exhibited the greatest antioxidant activity in both undigested and gastric‐digested PRx. However, tectoridin (P25) and quercetin (P40) only showed antioxidant activity at 1.616 ± 0.149 and 7.11 ± 3.19 mg TE/100 g dw, respectively, in the undigested phase. Antioxidant peaks of protocatechuic acid (P1) and tectoridin (P23) were retained only after gastric digestion, and no new quantifiable antioxidant peaks emerged after digestion.

**TABLE 3:** Effect of digestion on online 2,2′‐azino‐bis (3‐ethylbenzothiazoline‐6‐sulfonic acid free radical) (ABTS^•+^) antioxidant activity of phenolic compounds in grain extracts.

Peak	ABTS^•+^ antioxidant activity (mg TE/100 g dw)											
	Purple rice			Purple barley			Purple wheat			Blue Wheat		
	Undigested	Gastric	Intestinal	Undigested	Gastric	Intestinal	Undigested	Gastric	Intestinal	Undigested	Gastric	Intestinal
P1	3.62 ± 0.090a	4.26 ± 0.483a										
P2				4.28 ± 0.138a	1.29 ± 0.459b	1.12 ± 0.608b	2.26 ± 0.690a	1.962 ± 0.296b	1.087 ± 0.455b	3.063 ± 0.325a	1.28 ± 0.359b	1.145 ± 0.141b
P3				2.58 ± 0.297a								
Unknown (degradation product of P4)							3.925 ± 0.532			4.029 ± 0.661		
P8				57.8 ± 1.66								
P10				7.89 ± 0.316								
P12				18.15 ± 3.78								
P13	91.81 ± 0.642a	87.05 ± 4.51a		5.58 ± 0.942a	2.89 ± 0.764b							
P16	21.2 ± 5.57a	12.58 ± 0.805b										
P18				7.33 ± 0.676								
P23	1.92 ± 0.336a	1.35 ± 0.478a										
P25	1.616 ± 0.149											
P33												
P38									2.35 ± 0.449			2.057 ± 0.292
P40	7.11 ± 3.19											
P41				1.54 ± 0.200								

Abbreviations: –, not detected; **ABTS^•+^**, 2,2′‐azino‐bis (3‐ethylbenzothiazoline‐6‐sulfonic acid free radical); TE, Trolox equivalent.

*Note*: Data are the means ± SD (*n* = 3). Different alphabets in each row indicate a significant difference in antioxidant activity.

Similarly, in PBx, out of the eight antioxidant peaks detected in the undigested phase, only two peaks were detected in the gastric phase, and one was found in the intestinal phase. Cyanidin‐3‐glucoside (C3G) and P2 exhibited antioxidant activity at 2.89 ± 0.764 and 1.29 ± 0.459 mg TE/100 g in the gastric phase, while only P2 exhibited antioxidant activity at 1.12 ± 0.608 mg TE/100 g dw in the intestinal phase. The antioxidant activity of the compounds decreased significantly, and some compounds exhibited antioxidant activity only in the undigested phase.

The antioxidant activity profiles of both wheat extracts (PWx and BWx) were also significantly affected by digestion. Among the two antioxidant compounds, P2 and P3, only P2 showed antioxidant activity after gastric and intestinal digestion of the wheat extracts. P3 exhibited the greatest antioxidant activity in the undigested phase but did not recover after digestion. A new antioxidant peak attributed to P38 was detected in both extracts after intestinal digestion.

### 3.7 Caco‐2 cellular transport of polyphenols from digested extracts

Eleven compounds, mainly phenolic acids and flavones, were recovered from the basolateral chamber of the Caco‐2 cellular transport assay (Table 4). Among the transported compounds, four were recovered from PRx, six from PBx and PWx, and five from BWx. PCA was transported across the monolayer in all extracts, while P2 was transported from PBx, PWx, and BWx. Apigenin glycosides (P17, P20, and P21) were transported from both wheat extracts. Chrysoeriol (P43) was transported from PBx and PWx, but only in trace amounts from the latter.

**TABLE 4:** Recovery of phenolic compounds from the basolateral chamber after human colorectal adenocarcinoma cell line (Caco‐2) cell culture transport.

Compound	PRx compound recovery %	PBx compound recovery %	PWx compound recovery %	BWx compound recovery %
P1	48 ± 5bC	106 ± 4bA	104 ± 5bA	17 ± 21bD
P2		415 ± 32aA	292 ± 6aB	24 ± 9bC
P14	92 ± 6a			
P15		nil*		
P17			7 ± 1 dB	12 ± 6cA
P20			27 ± 2cA	37 ± 12bA
P21			4 ± 0eB	100 ± 8aA
P25	92 ± 4a			
P26	23 ± 19b			
P33		3.2 ± 6c		
P43		119 ± 11b	Trace	

*Note*: Data are presented as mean ± SD (*n* = 3). Different lowercase letters in a column indicate a significant difference in the percentage recovery within an extract. Different uppercase letters in each row indicate significant differences in the percentage recovery between the extracts for the same compound. The percentage recovery was calculated using the concentration of compounds introduced into the apical chamber against the concentration of compounds in the basolateral layer.

Abbreviations: PRx, purple rice extract; PBx, purple barley extract; PWx, purple wheat extract, PWx.

The percentage of compounds recovered in the basolateral layer was calculated to determine the proportion of compounds transported from the extract. Some phenolic compounds showed relatively high recovery. Among the PRx compounds, vanillic acid (P14) and tectoridin (P25) showed the highest recoveries, with each having approximately 92% recovery. Among the PBx compounds, chrysoeriol and P2 had the highest recoveries of 119% and 419%, respectively. P2 had the highest recovery from PWx at 282%. Among the BWx compounds, apigenin‐6‐C‐arabinoside‐8‐C‐hexoside isomer 2 (P21) had the highest recovery at 100%. P2 demonstrated a higher recovery when transported from PBx compared to the wheat extracts, whereas apigenin‐6‐C‐arabinoside‐8‐C‐hexoside isomer 2 (P21) showed a higher recovery from BWx than from PWx. Chrysoeriol (P43) showed a higher recovery when transported from PBx than from PWx.

Most of the compounds introduced into the apical chamber were detected in the basolateral chamber. Only one compound, P15, derived from PBx, was found in the apical chamber but not in the basolateral chamber. In addition, some compounds detected after digestion were not detected after dilution with the media (Tables 2 and 4).

## 4 DISCUSSION

This study aimed to determine the impact of simulated digestion and intestinal cellular transport on the phenolic composition and antioxidant potential of pigmented grain extracts (PRx, PBx, PWx, and BWx). These pigmented grain extracts were shown in our previous study to be rich in bioactive polyphenols, but the compounds needed to resist digestive degradation to be absorbed into the bloodstream to exert their health effects in vivo (Ed Nignpense et al., 2022b). The results of this study showed that the antioxidant activity measured using FRAP and ABTS^•+^ significantly decreased in all extracts after digestion, with the largest decrease observed in PRx (Figures 2 and 3). However, the decrease in antioxidant activity was not proportional to the observed decrease in total phenolic content, indicating that other factors, such as the breakdown of the chemical structure of compounds, may contribute to the observed decrease in antioxidant activity (Ed Nignpense et al., 2022b; Sun et al., 2015). Caco‐2 cellular transport experiments showed that some compounds, such as P2 and apigenin‐6‐C‐arabinoside‐8‐C‐hexoside isomer 2, are transported differently among the cereal extracts, indicating that the composition of extracts may influence their bioavailability (Table 4).

**FIGURE 1:**
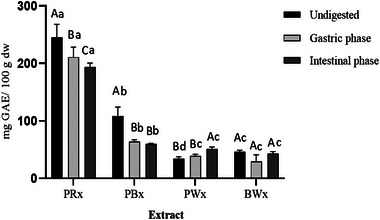
Changes in the total phenolic content (determined by the Folin–Ciocalteu method) of pigmented grain phenolic extracts before and after simulated gastrointestinal digestion. Data are blank corrected, expressed as mg GAE/100 g dry weight flour, and represent mean ± SD; *n* = 3. Significant differences within and between grain extracts are represented by different capital letters and lowercase letters, respectively. PRx, purple rice extract; PBx, purple barley extract; PWx, purple wheat extract; BWx, blue wheat extract.

The impact of in vitro digestion on the total phenolic content of pigmented grain phenolic extracts was notable, with extracts PRx and PBx showing a decrease in phenolic content. This is consistent with a previous study by Villalva et al. (2022), which indicated that phenolic compounds can be degraded in the digestive medium. However, the degree of decrease in phenolic content was not uniform across the extracts, suggesting that the phenolic compounds present in each extract have different susceptibilities to digestion (Figure 1). The results indicated that the undigested PRx and PBx extracts had a higher concentration of anthocyanins and flavan‐3‐ols, leading to a greater overall phenolic content compared to PWx and BWx (Figure 1; Table 2). The loss of these compounds during digestion resulted in a significant reduction in the total phenolic content of PRx and PBx. Although anthocyanins and flavan‐3‐ols are known to convert or hydrolyze into chalcones and phenolic acids under alkaline conditions, the stability of flavonoid glycosides may have contributed to the maintenance of the total phenolic content during digestion (Yang et al., 2018) (Table 2). PWx and BWx had similar digestion profiles which could partly be attributed to the stability of their flavonoid glycosides. Interestingly, PWx showed a slight but significant increase in phenolic content (126%) after digestion, possibly due to phenolic metabolites or non‐phenolic compounds, such as tryptophan, present in the digesta. Moreover, the polyphenol metabolites produced during digestion have antioxidant potential and may show reactivity against Folin–Ciocalteu, due to the observed increase in total phenolic content and antioxidant activity after digestion (Tarko et al., 2020).

**FIGURE 2:**
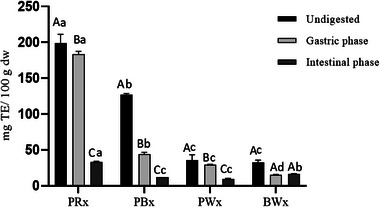
Changes in the total antioxidant activity (determined by ferric reducing antioxidant power [FRAP] assay) of pigmented grain phenolic extracts before and after simulated gastrointestinal digestion. Data are blank corrected, expressed as mg TE/100 g dry weight flour, and represent mean ± SD; *n* = 3. Significant differences within and between grain extracts are represented by different capital letters and lowercase letters, respectively. PRx, purple rice extract; PBx, purple barley extract; PWx, purple wheat extract; BWx, blue wheat extract; TE, Trolox equivalent.

**FIGURE 3:**
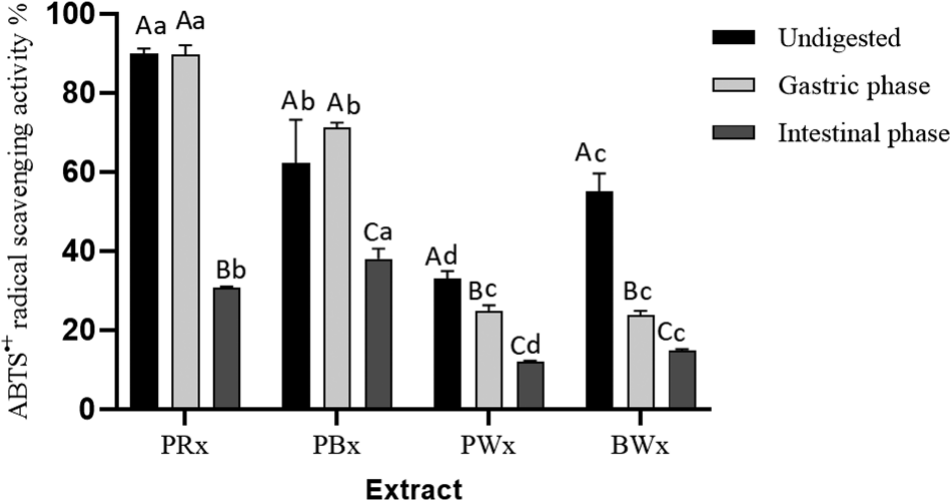
Changes in the total 2,2′‐azino‐bis (3‐ethylbenzothiazoline‐6‐sulfonic acid free radical) (ABTS^•+^) radical scavenging activity of pigmented grain phenolic extracts before and after simulated gastrointestinal digestion. Data are blank corrected, expressed as mg TE/100 g dry weight flour, and represent mean ± SD; *n* = 3. Significant differences within and between grain extracts are represented by different capital letters and lowercase letters, respectively. PRx, purple rice extract; PBx, purple barley extract; PWx, purple wheat extract; BWx, blue wheat extract.

After digestion of the grain extracts, only a small proportion of total antioxidant activity was retained (Figures 2 and 3). The FRAP and ABTS^•+^ antioxidant activities significantly decreased after complete digestion, with the largest decrease observed in PRx and PBx. This decrease in antioxidant activity can be attributed to the reduction in metal‐chelating and radical‐scavenging compounds, all of which are known to contribute to the total antioxidant activity of phenolic extracts. Interestingly, although the ABTS^•+^ antioxidant activity of PRx was initially higher than that of PRx, after digestion, PRx had a lower ABTS^•+^ antioxidant activity. This decrease in antioxidant activity may be due to the fragmentation of phenolic compounds, such as anthocyanins in PRx, which affects their ability to scavenge free radicals. This finding is consistent with a study by David et al. (2019), which demonstrated a dramatic reduction in ABTS^•+^ scavenging activity after digestion of anthocyanin‐rich cornelian cherry extracts. Nevertheless, other phenolic compounds, such as flavonol glycosides, which decreased in quantity in the digestion profiles of PWx and BWx, can also contribute to the reduction in total antioxidant activity of extracts (Tables 1 and 2; Table S1).

Comparing the two phases of digestion, grain extracts retained more antioxidant activity in the gastric phase than in the intestinal phase, owing to the stability of some antioxidant phenolic compounds at acidic pH. This suggests that extracts may exert antioxidant activity primarily at the gastric level (Gawlik‐Dziki et al., 2009). The changes observed in the benchtop antioxidant assays were comparable to the phenolic profiles generated from the LC‐QTOF‐MS analysis (Tables 2 and 3). The recovery of fewer phenolic compounds after digestion likely resulted in a decrease in the total phenolic content. Anthocyanins, C3G (13.84 ± 0.106 GAE/100 g dw) and peonidin‐3‐glucoside (P3G, 5.61 ± 0.227 GAE/100 g dw), recovered after gastric digestion would have greatly contributed to the total phenolic content in this phase. The loss of compound flavan‐3‐ols, such as prodelphinidin B3, catechin, and procyanidin B3, occurring after gastric digestion led to a decrease in the total phenolic content (Figure 1; Table 2). The emergence of new compounds P5, P6, P37, and P38 after intestinal digestion may have slightly increased the total phenolic content; however, the loss of anthocyanins caused a greater reduction in the total phenolic content, FRAP values, and ABTS^•+^ antioxidant activity. However, anthocyanin stability is important owing to its reported health benefits. Thus, food technologies such as microencapsulation that improve the stability of anthocyanins and other bioactive compounds in extracts should be employed to improve the bioaccessibility and bioavailability of polyphenols (Peanparkdee et al., 2021).

The changes observed in total ABTS^•+^ activity can be attributed to the ABTS^•+^ antioxidant profile of the digested extracts. The decrease in the number of antioxidant active peaks after digestion concurred with the benchtop results, indicating a decrease in total ABTS^•+^ antioxidant activity (Figure 3 and Table 3). Interestingly, P2 was the only antioxidant‐active compound detected before and after gastric and intestinal digestion of the barley and wheat extracts (Table 3). This may be due to the ability of the compound to maintain the chemical structure for free radical scavenging activity under digestive conditions. Although the compound's antioxidant activity was significantly reduced in the extracts, it most likely contributed to the total ABTS^•+^ activity measured. As expected, anthocyanins (C3G and P3G) were recovered after gastric digestion and were the main contributors to the total radical scavenging activities of PRx and PBx (Figure 3 and Table 3). It is also noteworthy that digested PRx retained approximately 31% of its total ABTS activity, although it did not show any quantifiable ABTS^•+^ antioxidant peaks after intestinal digestion. This was likely due to the cumulative and synergistic antioxidant activity of the recovered phenolic compounds after digestion (Table 2) (Skroza et al., 2022).

The results of Caco‐2 cellular transport experiments demonstrated the transport of metabolites derived from digestion and variations in the efficiency of transport of phenolic compounds within and between grain phenolic extracts. Specifically, phenolic acids and flavone glycosides were the primary compounds transported, as indicated in Table 4. Caco‐2 cellular transport experiments revealed differences in the transport efficacy of phenolic compounds within and between grain phenolic extracts, with phenolic acids and flavone glycosides being the main compounds transported (Table 4). No new phenolic metabolites were detected from the Caco‐2 transport. However, compounds found in metabolic processes in enterocytes include glycosylated phenolic compounds (Li et al., 2020). Interestingly, among the digestion metabolites, namely, vanillic acid, P5, and P38, only vanillic acid was transported across the monolayer. This is likely due to the relatively small size of the vanillic acid (Table 1). Protocatechuic acid was the only compound transported across all extracts, but it showed significantly greater transport efficacy in PBx and PWx than in other extracts. Chrysoeriol was only transported from both PBx and PWx but was found in trace amounts in the latter. These results suggest possible differences in matrix‐dependent transport efficacy, similar to findings from the study by Hilary et al. (2020), which showed differences in transport efficacy between different forms of date seeds. The disparities in transport efficiency between phenolic compounds can be attributed to the chemical structure and solubility of the compounds as well as differences in the transport mechanism (Fang et al., 2017; Marina et al., 2019). The transportation of simple phenolic acids, such as protocatechuic acid and vanillic acid, and flavonoid aglycones, such as chrysoeriol, is likely facilitated by direct passive diffusion. In contrast, the transport of flavonoid glycosides requires the active assistance of glucose transporters (Li et al., 2020). Among the polyphenols transported across the monolayer, there were more flavonoid glycosides than phenolic acids detected (Table 4). Nonetheless, further research will be necessary to accurately determine the mode of cellular transport by compounds, particularly concerning unknowns. Compounds that are actively transported will provide insight into the mechanisms involved in cellular processes and may aid in identifying potential targets for improving bioavailability in future studies. Overall, these findings underscore the importance of considering the food matrix when assessing the bioavailability of health‐promoting polyphenols. However, it should be noted that the in vitro nature of the experiment does not fully reflect the in vivo dynamics of absorption. However, the use of both grain polyphenol extracts and polyphenol‐fortified grain foods in future in vivo studies will provide a comprehensive understanding of the bioavailability and bioefficacy of polyphenols in isolation as well as within a complex food matrix.

## 5 CONCLUSION

In conclusion, this study offers valuable insights into the changes in the phenolic composition and antioxidant activity of pigmented grain extracts after simulated digestion and Caco‐2 cellular transport. The antioxidant activity of all extracts was significantly impacted by digestion, with the largest decrease in antioxidant activity observed in purple rice extract and the least decrease observed in the pigmented wheat extracts. Additionally, the degree of decrease in phenolic content was not uniform across extracts due to the difference in susceptibilities that compounds had to digestive degradation. The breakdown of anthocyanins and flavan‐3‐ols from PRx and PBx was associated with reduced total phenolic content, FRAP values, and ABTS^•+^ antioxidant activity. The Caco‐2 transport experiment revealed that phenolic acids and flavone glycosides from extracts were likely absorbed by passive or active transport, with transport efficiency being matrix‐dependent. Consequently, future studies should investigate food processing techniques and microencapsulation technologies to improve the stability and absorption of the antioxidant compounds. Finally, future human dietary intervention studies will be required to determine the bioavailability and beneficial health effects of these grain polyphenols in vivo.

## AUTHOR CONTRIBUTIONS

**Borkwei Ed Nignpense**: Conceptualization; methodology; formal analysis; writing—original draft; writing—review and editing; investigation. **Nidhish Francis**: Conceptualization; validation; supervision; writing—review and editing. **Christopher Blanchard**: Conceptualization; validation; writing—review and editing; supervision; project administration; funding acquisition; resources. **Abishek Bommannan Santhakumar**: Conceptualization; writing—review and editing; supervision; validation; project administration; funding acquisition.

## CONFLICT OF INTEREST STATEMENT

The authors declare no conflict of interest.

## Supporting information

Supporting Information

## Data Availability

Data will be available on request.

## References

[jfds17009-bib-0001] Callcott, E. T. , Thompson, K. , Oli, P. , Blanchard, C. L. , & Santhakumar, A. B. (2018). Coloured rice‐derived polyphenols reduce lipid peroxidation and pro‐inflammatory cytokines ex vivo. Food and Function, 9(10), 5169–5175. 10.1039/c8fo01531g 30255188

[jfds17009-bib-0002] D'Archivio, M. , Filesi, C. , Varì, R. , Scazzocchio, B. , & Masella, R. (2010). Bioavailability of the polyphenols: Status and controversies. International Journal of Molecular Sciences, 11(4), 1321–1342. 10.3390/ijms11041321 20480022 PMC2871118

[jfds17009-bib-0003] David, L. , Danciu, V. , Moldovan, B. , & Filip, A. (2019). Effects of in vitro gastrointestinal digestion on the antioxidant capacity and anthocyanin content of cornelian cherry fruit extract. Antioxidants, 8(5), 114. 10.3390/antiox8050114 31052224 PMC6562851

[jfds17009-bib-0004] Drawbridge, P. C. , Apea‐Bah, F. , Silveira Hornung, P. , & Beta, T. (2021). Bioaccessibility of phenolic acids in Canadian hulless barley varieties. Food Chemistry, 358, 129905. 10.1016/j.foodchem.2021.129905 33940288

[jfds17009-bib-0005] Ed Nignpense, B. , Chinkwo, K. A. , Blanchard, C. L. , & Santhakumar, A. B. (2020). Black sorghum phenolic extract modulates platelet activation and platelet microparticle release. Nutrients, 12(6), 1760. 10.3390/nu12061760 32545505 PMC7353362

[jfds17009-bib-0006] Ed Nignpense, B. , Francis, N. , Blanchard, C. , & Santhakumar, A. B. (2021). Bioaccessibility and bioactivity of cereal polyphenols: A review. Foods, 10(7), 1595. 10.3390/foods10071595 34359469 PMC8307242

[jfds17009-bib-0007] Ed Nignpense, B. , Latif, S. , Francis, N. , Blanchard, C. , & Santhakumar, A. B. (2022a). Bioaccessibility and antioxidant activity of polyphenols from pigmented barley and wheat. Foods, 11(22), 3697. 36429289 10.3390/foods11223697 PMC9689394

[jfds17009-bib-0008] Ed Nignpense, B. , Latif, S. , Francis, N. , Blanchard, C. , & Santhakumar, A. B. (2022b). The impact of simulated gastrointestinal digestion on the bioaccessibility and antioxidant activity of purple rice phenolic compounds. Food Bioscience, 47, 101706. 10.1016/j.fbio.2022.101706

[jfds17009-bib-0009] Fang, Y. , Cao, W. , Xia, M. , Pan, S. , & Xu, X. (2017). Study of structure and permeability relationship of flavonoids in Caco‐2 cells. Nutrients, 9(12), 1301. 10.3390/nu9121301 29186068 PMC5748751

[jfds17009-bib-0010] Francis, N. , Rao, S. , Blanchard, C. , & Santhakumar, A. (2019). Black sorghum phenolic extract regulates expression of genes associated with oxidative stress and inflammation in human endothelial cells. Molecules, 24(18), 3321. 10.3390/molecules24183321 31547324 PMC6767043

[jfds17009-bib-0011] Gamel, T. H. , Wright, A. J. , Tucker, A. J. , Pickard, M. , Rabalski, I. , Podgorski, M. , Di Ilio, N. , O'Brien, C. , & Abdel‐Aal, E.‐S. M. (2019). Absorption and metabolites of anthocyanins and phenolic acids after consumption of purple wheat crackers and bars by healthy adults. Journal of Cereal Science, 86, 60–68. 10.1016/j.jcs.2018.11.017

[jfds17009-bib-0012] Gawlik‐Dziki, U. , Dziki, D. , Baraniak, B. , & Lin, R. (2009). The effect of simulated digestion in vitro on bioactivity of wheat bread with Tartary buckwheat flavones addition. LWT—Food Science & Technology, 42(1), 137–143. 10.1016/j.lwt.2008.06.009

[jfds17009-bib-0013] Hilary, S. , Tomás‐Barberán, F. A. , Martinez‐Blazquez, J. A. , Kizhakkayil, J. , Souka, U. , Al‐Hammadi, S. , Habib, H. , Ibrahim, W. , & Platat, C. (2020). Polyphenol characterisation of *Phoenix dactylifera* L. (date) seeds using HPLC‐mass spectrometry and its bioaccessibility using simulated in‐vitro digestion/Caco‐2 culture model. Food Chemistry, 311, 125969. 10.1016/j.foodchem.2019.125969 31864186

[jfds17009-bib-0014] Li, S. , Liu, J. , Li, Z. , Wang, L. , Gao, W. , Zhang, Z. , & Guo, C. (2020). Sodium‐dependent glucose transporter 1 and glucose transporter 2 mediate intestinal transport of quercetrin in Caco‐2 cells. Food & Nutrition Research, 64, 00–00. 10.29219/fnr.v64.3745 PMC7307431 32612490

[jfds17009-bib-0015] Liu, Q. , Qiu, Y. , & Beta, T. (2010). Comparison of antioxidant activities of different colored wheat grains and analysis of phenolic compounds. Journal of Agricultural and Food Chemistry, 58(16), 9235–9241. 10.1021/jf101700s 20669971

[jfds17009-bib-0016] Manach, C. , Williamson, G. , Morand, C. , Scalbert, A. , & Rémésy, C. (2005). Bioavailability and bioefficacy of polyphenols in humans. I. Review of 97 bioavailability studies. American Journal of Clinical Nutrition, 81(1), 230S–242S. 10.1093/ajcn/81.1.230S 15640486

[jfds17009-bib-0017] Marina, Z. , Amin, I. , Loh, S. , Fadhilah, J. , & Kassim, K. (2019). Intestinal permeability and transport of apigenin across caco‐2 cell monolayers. Journal of Food Bioactives, 7, 00–00. 10.31665/JFB.2019.7198

[jfds17009-bib-0018] Peanparkdee, M. , Borompichaichartkul, C. , & Iwamoto, S. (2021). Bioaccessibility and antioxidant activity of phenolic acids, flavonoids, and anthocyanins of encapsulated Thai rice bran extracts during in vitro gastrointestinal digestion. Food Chemistry, 361, 130161. 10.1016/j.foodchem.2021.130161 34051598

[jfds17009-bib-0019] Rao, S. , Callcott, E. T. , Santhakumar, A. B. , Chinkwo, K. A. , Vanniasinkam, T. , Luo, J. , & Blanchard, C. L. (2018). Profiling polyphenol composition and antioxidant activity in Australian‐grown rice using UHPLC Online‐ABTS system. Journal of Cereal Science, 80, 174–179. 10.1016/j.jcs.2018.02.011

[jfds17009-bib-0020] Rao, S. , Chinkwo, K. , Santhakumar, A. , Johnson, S. , & Blanchard, C. (2019). Apoptosis induction pathway in human colorectal cancer cell line SW480 exposed to cereal phenolic extracts. Molecules, 24(13), 2465. 10.3390/molecules24132465 31277499 PMC6651285

[jfds17009-bib-0021] Saji, N. , Schwarz, L. J. , Santhakumar, A. B. , & Blanchard, C. L. (2020). Stabilization treatment of rice bran alters phenolic content and antioxidant activity. Cereal Chemistry, 97(2), 281–292. 10.1002/cche.10243

[jfds17009-bib-0022] Shen, Y. , Zhang, H. , Cheng, L. , Wang, L. , Qian, H. , & Qi, X. (2016). In vitro and in vivo antioxidant activity of polyphenols extracted from black highland barley. Food Chemistry, 194, 1003–1012. 10.1016/j.foodchem.2015.08.083 26471646

[jfds17009-bib-0023] Skroza, D. , Šimat, V. , Vrdoljak, L. , Jolić, N. , Skelin, A. , Čagalj, M. , Frleta, R. , & Generalić Mekinić, I. (2022). Investigation of antioxidant synergisms and antagonisms among phenolic acids in the model matrices using FRAP and ORAC methods. Antioxidants, 11(9), 1784. 10.3390/antiox11091784 36139858 PMC9495677

[jfds17009-bib-0024] Sun, D. , Huang, S. , Cai, S. , Cao, J. , & Han, P. (2015). Digestion property and synergistic effect on biological activity of purple rice (*Oryza sativa* L.) anthocyanins subjected to a simulated gastrointestinal digestion in vitro. Food Research International, 78, 114–123. 10.1016/j.foodres.2015.10.029 28433272

[jfds17009-bib-0025] Tarko, T. , Duda‐Chodak, A. , & Soszka, A. (2020). Changes in phenolic compounds and antioxidant activity of fruit musts and fruit wines during simulated digestion. Molecules, 25(23), 5574. 33260996 10.3390/molecules25235574 PMC7730555

[jfds17009-bib-0026] Villalva, M. , Jaime, L. , Siles‐Sánchez, M. D. , & Santoyo, S. (2022). Bioavailability assessment of yarrow phenolic compounds using an in vitro digestion/Caco‐2 cell model: Anti‐inflammatory activity of basolateral fraction. Molecules, 27(23), 8254. 36500344 10.3390/molecules27238254 PMC9740014

[jfds17009-bib-0027] Yang, P. , Yuan, C. , Wang, H. , Han, F. , Liu, Y. , Wang, L. , & Liu, Y. (2018). Stability of anthocyanins and their degradation products from Cabernet Sauvignon red wine under gastrointestinal ph and temperature conditions. Molecules, 23(2), 354. 10.3390/molecules23020354 29414926 PMC6017626

