# Supplementary material for: Effect of gastrointestinal digestion on the stability, antioxidant activity, and Caco‐2 cellular transport of pigmented grain polyphenols
Source: J Food Sci. 2024 Mar 11;89(5):2701–15. doi: 10.1111/1750-3841.17009 (PMC13281133; doi:10.1111/1750-3841.17009)
Supplement: Supplementary file 1 — Supporting Information [file JFDS-89-2701-s001.docx]

# Supplementary Materials

**Effect of gastrointestinal digestion on the stability, antioxidant activity and Caco-2 cellular transport of pigmented grain polyphenols**

1. Undigested PRx


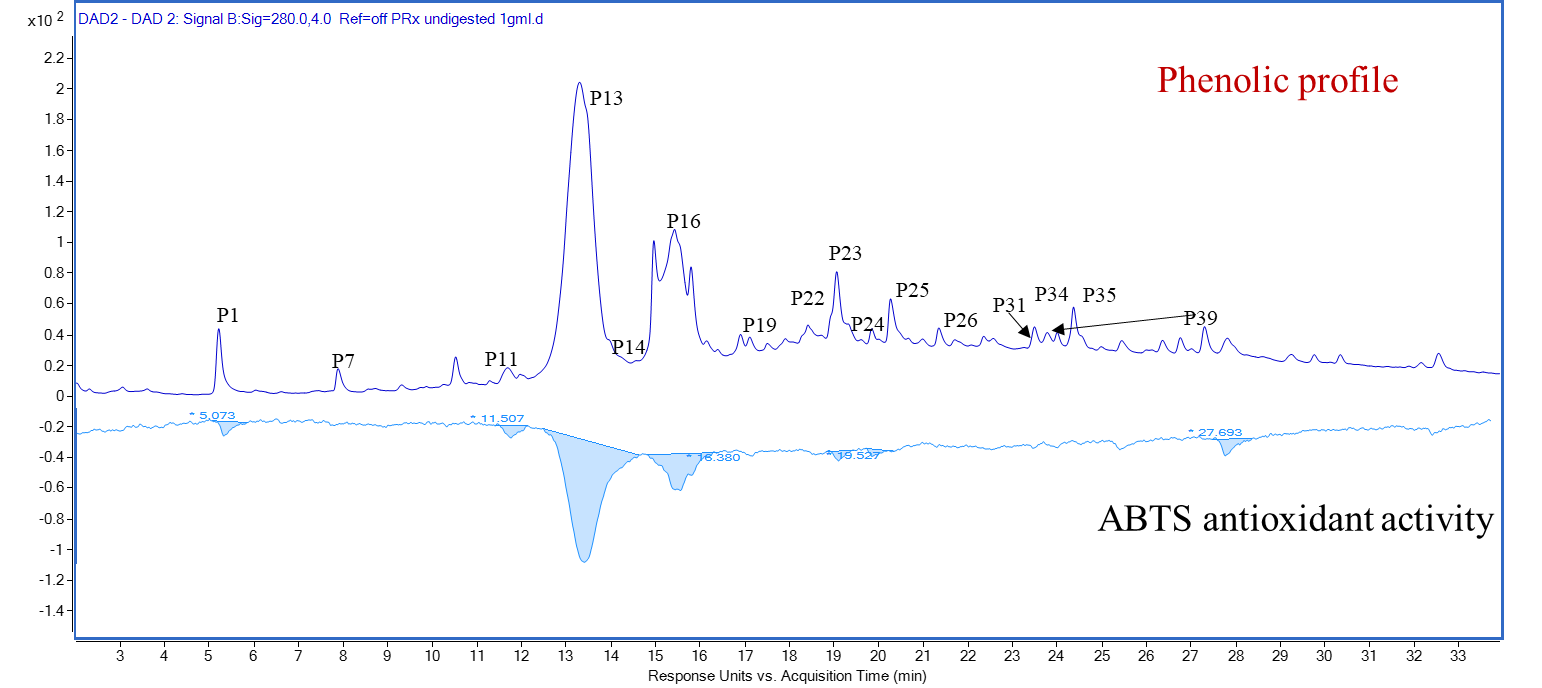


1. Gastric digested PRx


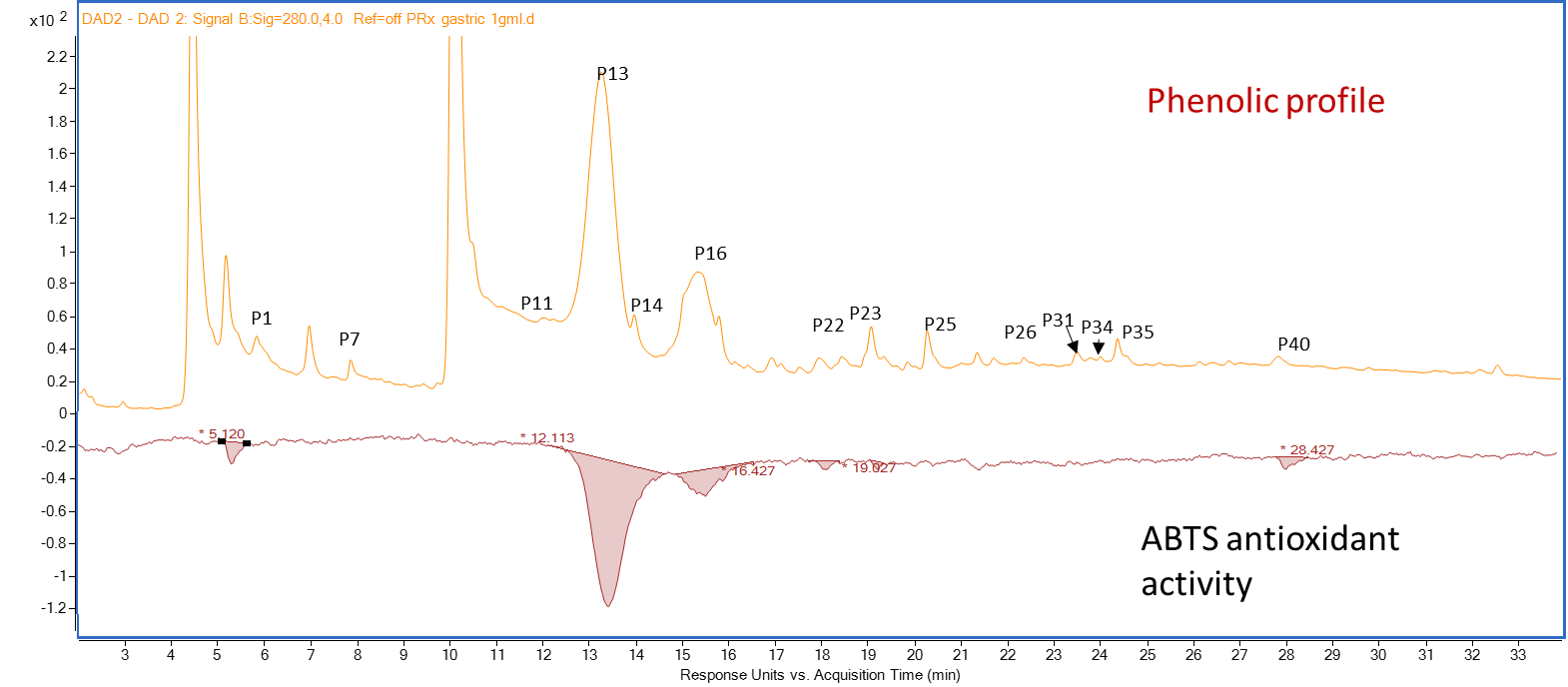


1. Intestinal digested PRx


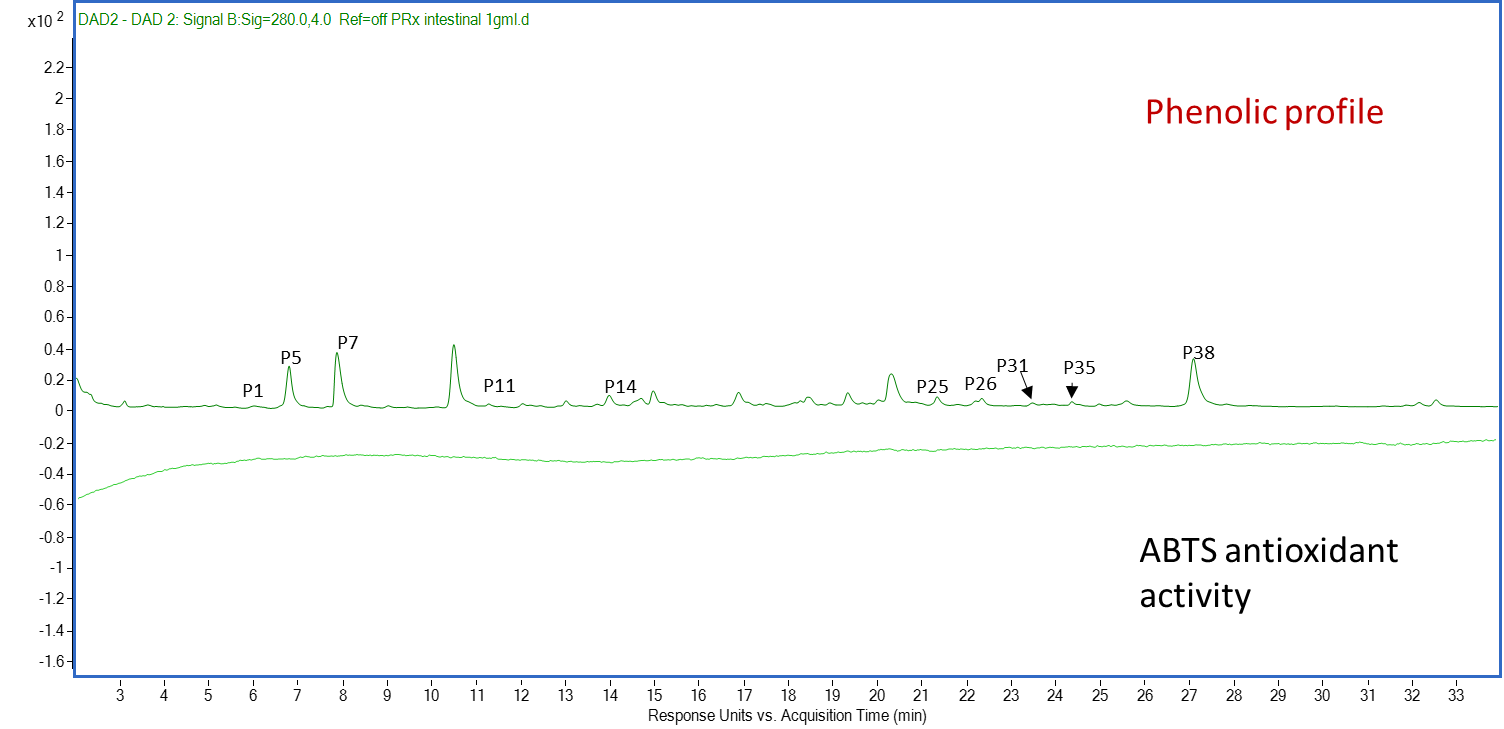


P9

**Figure S1.** UHPLC-ABTS antioxidant profile of PRx before and after digestion

1. Undigested PBx


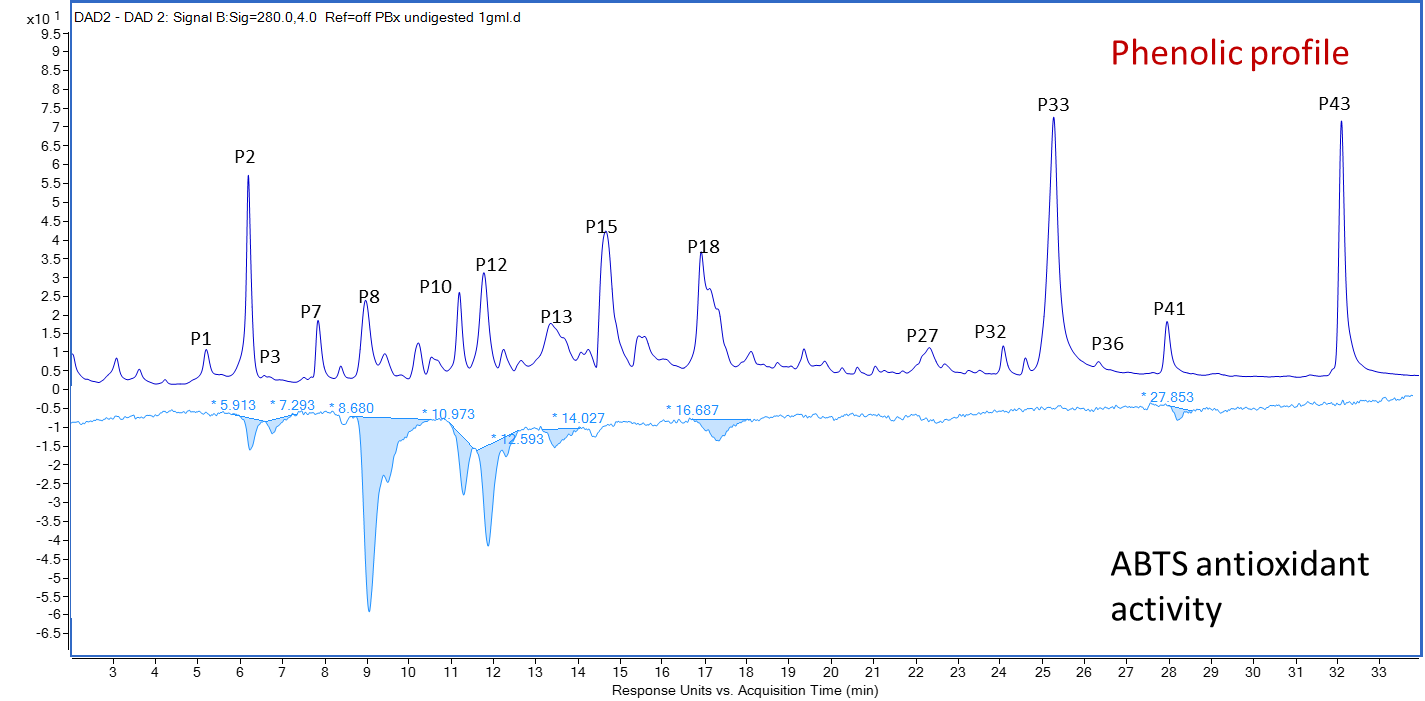


1. Gastric digested PBx


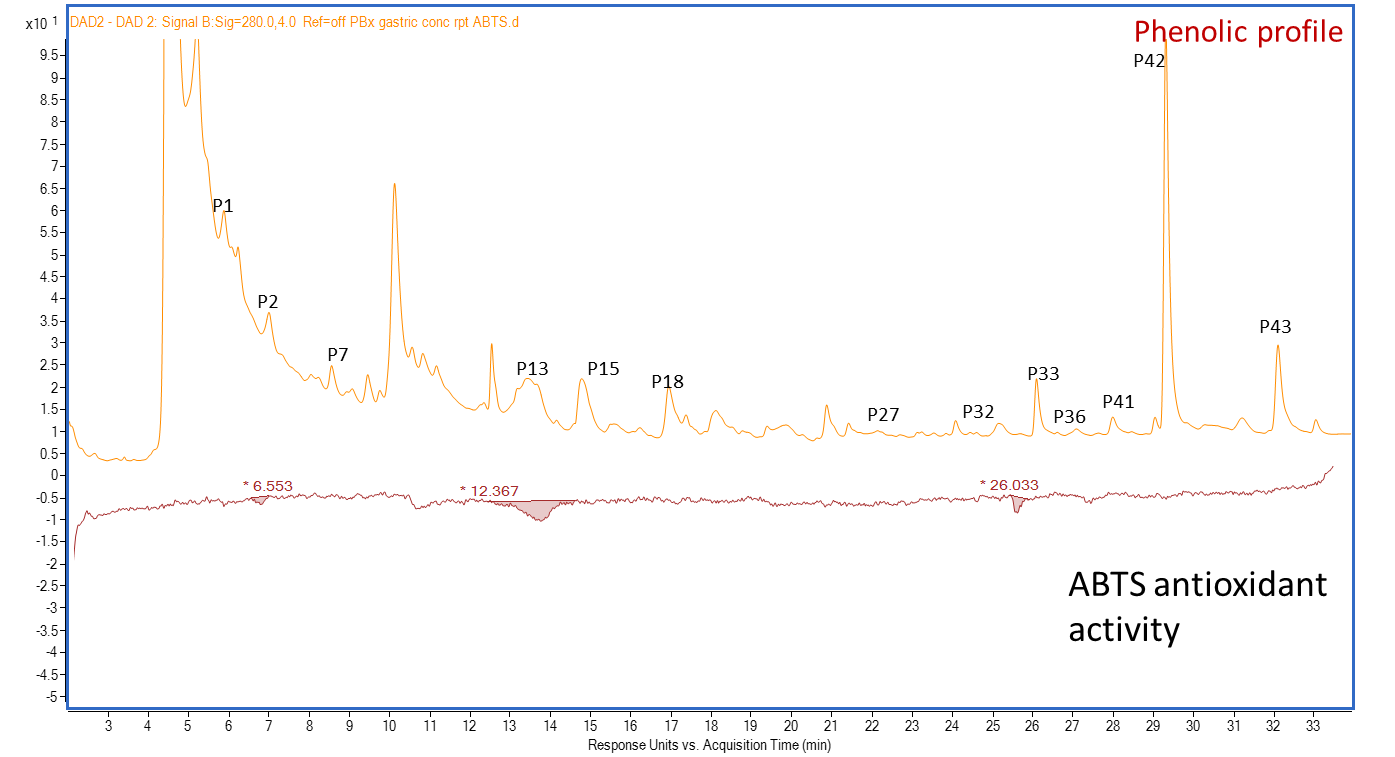


1.
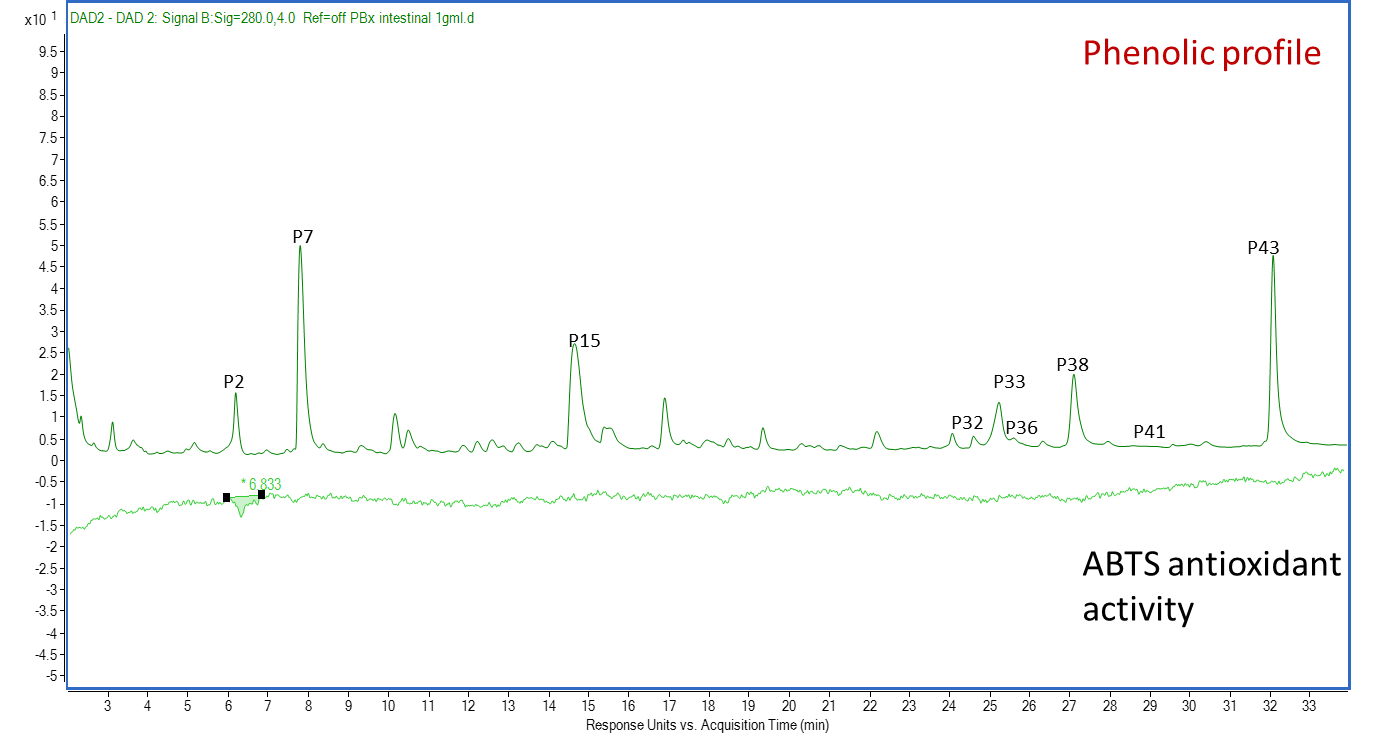
Intestinal digested PBx

**Figure S2.** UHPLC-ABTS antioxidant profile of PBx before and after digestion

1. Undigested PWx


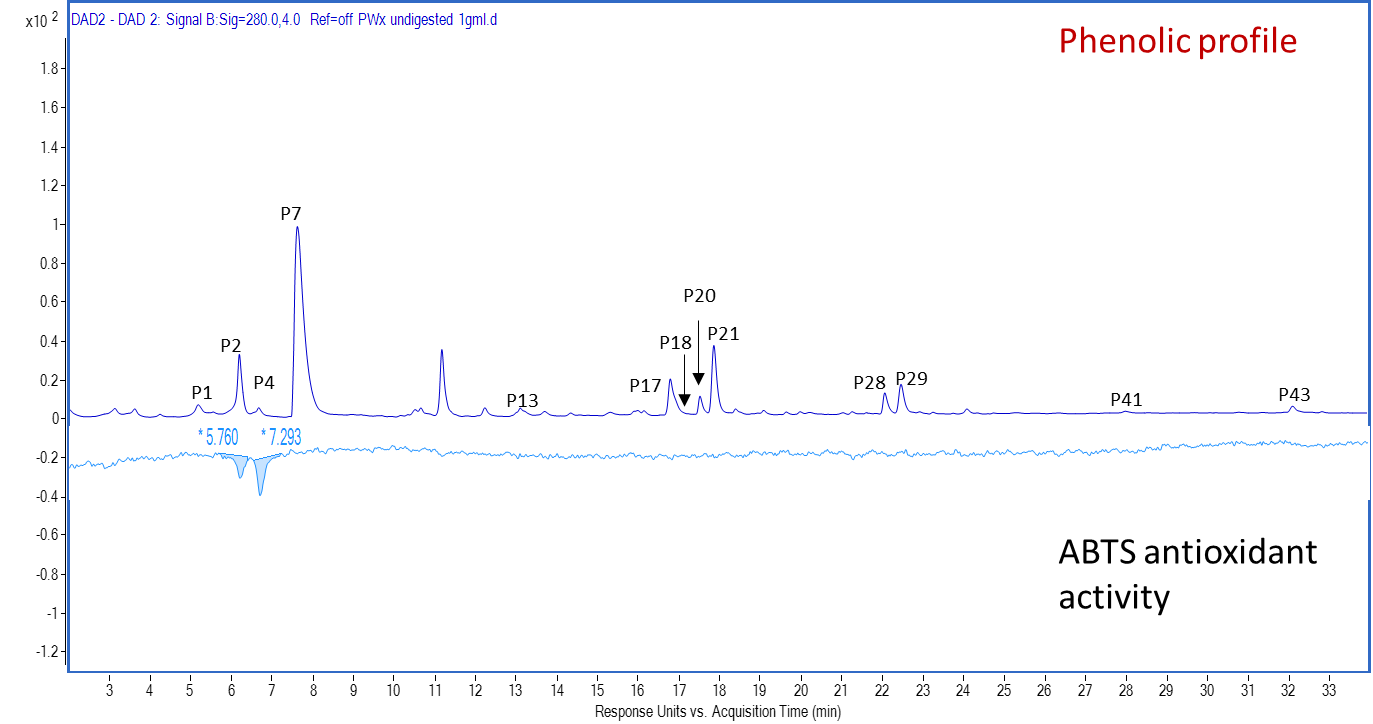


1. Gastric digested PWx


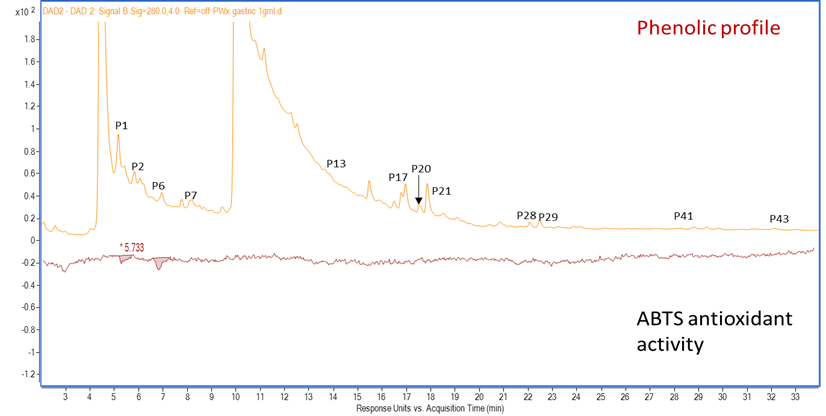


1. Intestinal digested PWx


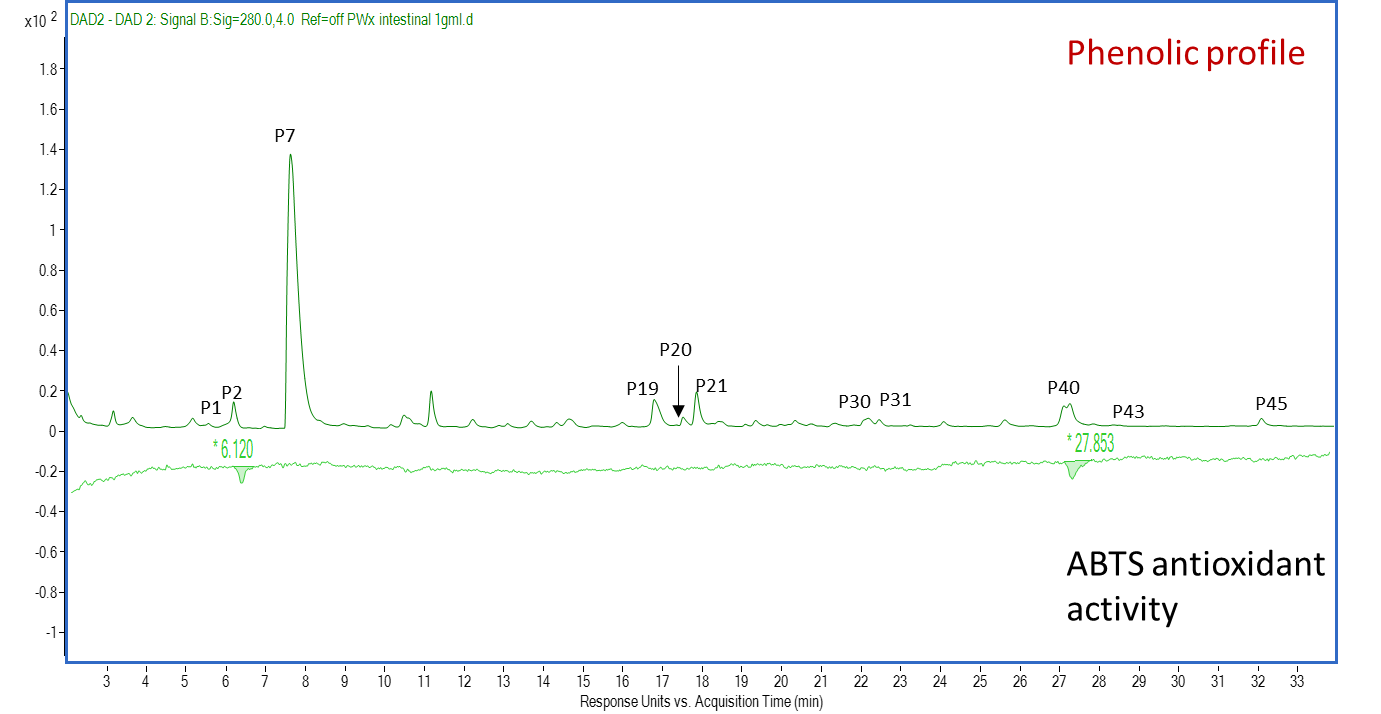


**Figure S3.** UHPLC-ABTS antioxidant profile of PWx before and after digestion

1. Undigested BWx


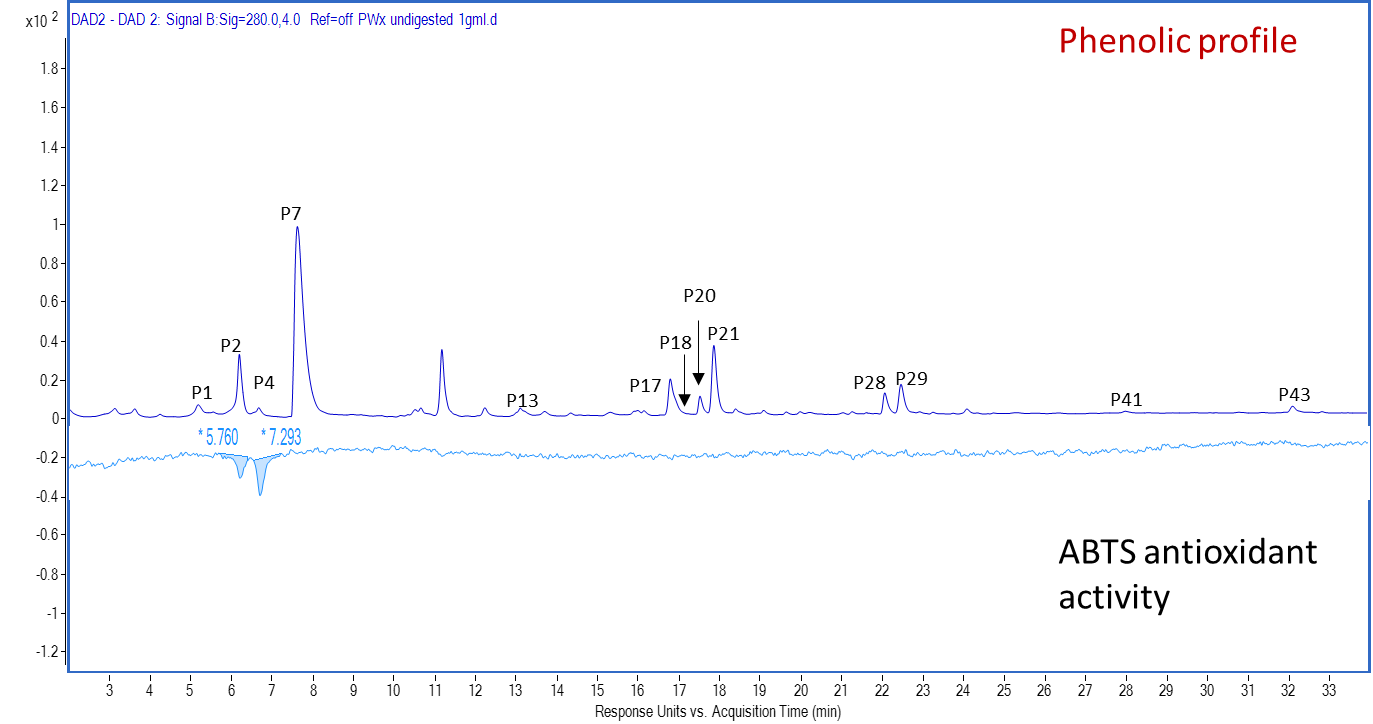


1. Gastric digested BWx


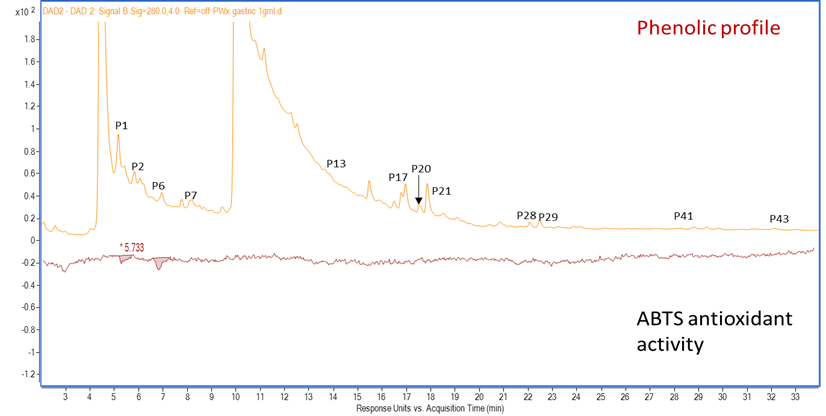


1. Intestinal digested BWx


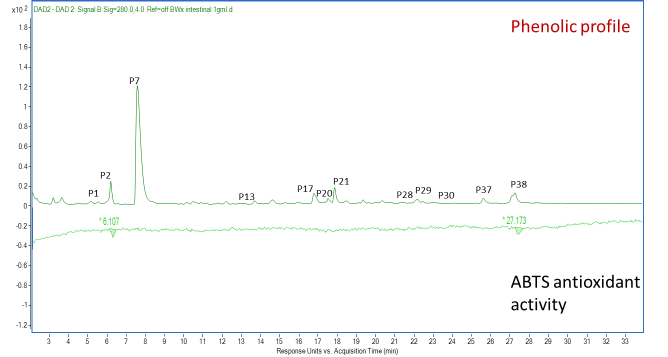


**Figure S4.** UHPLC-ABTS antioxidant profile of BWx before and after digestion

**Table S1** Changes to the levels of the main polyphenol classes following in vitro digestion of extracts.

| **Polyphenol Class** | **PRx** | | | **PBx** | | | **PWx** | | | | **BWx** | | | |
| --- | --- | --- | --- | --- | --- | --- | --- | --- | --- | --- | --- | --- | --- | --- |
|  | **Undigested** | **Gastric** | **Intestinal** | **Undigested** | **Gastric** | **Intestinal** | **Undigested** | **Gastric** | **Intestinal** | **Undigested** | | **Gastric** | **Intestinal** |  |
| Phenolic acids | 1.16 | 2.378 | 0.195 | 0.175 | 0.266 | 0 | 0.144 | 0.897 | 0.098 | 0.097 | | 0.394 | 0.059 |  |
| Anthocyanins | 9.43 | 5.61 | 0 | 1.975 | 0.425 | 0 | 0 | 0 | 0 | 0 | | 0 | 0 |  |
| Flavan-3-ols | 0 | 0 | 0 | 2.459 | 0 | 0 | 0 | 0 | 0 | 0 | | 0 | 0 |  |
| Flavones & glycosides | 0 | 0 | 0 | 6.086 | 1.065 | 1.499 | 2.021 | 1.815 | 1.06 | 2.404 | | 0.999 | 0.769 |  |
| Flavanols & glycosides | 2.311 | 1.021 | 0.093 | 0 | 0 | 0 | 0 | 0 | 0 | 0 | | 0 | 0 |  |
| Total (mg GAE/100g dw) | 12.901 | 9.009 | 0.288 | 10.695 | 1.756 | 1.499 | 2.165 | 2.712 | 1.158 | 2.501 | | 1.393 | 0.828 |  |

**Table S2** Concentration of digested grain extracts compounds detected in Caco-2 culture media from both apical and basolateral chambers.

| **Peak** | **PRx**  **(mg GAE/100g dw)** | | | **PBx**  **(mg GAE/100g dw)** | | | **PWx**  **(mg GAE/100g dw)** | | | **BWx**  **(mg GAE/100g dw)** | | |
| --- | --- | --- | --- | --- | --- | --- | --- | --- | --- | --- | --- | --- |
|  | **Amount introduced** | **Apical** | **Basolateral** | **Amount introduced** | **Apical** | **Basolateral** | **Amount introduced** | **Apical** | **Basolateral** | **Amount introduced** | **Apical** | **Basolateral** |
| P1(Protocatechuic acid) | 0.192 ± 0.028a | 0.192 ± 0.034a | 0.0930 ± 0.010b | 0.259 ± 0.006a | 0.272 ± 0.002a | 0.276 ±0.011a | 0.259 ± 0.020a | 0.279 ± 0.019a | 0.270 ± 0.014a | 0.168 ± 0.028a | 0.171 ± 0.014a | 0.201 ± 0.036a |
| P2 |  |  |  | 0.176 ± 0.006c | 0.239 ± 0.017b | 0.732 ±0.057a | 0.239 ± 0.003c | 0.508 ± 0.015b | 0.699 ± 0.015a | 0.664 ± 0.035a | 0.515 ± 0.083b | 0.157 ± 0.062c |
| P14 (Vanillic acid) | 0.170 ± 0.011a | 0.119 ± 0.005b | 0.157 ± 0.011a |  |  |  |  |  |  |  |  |  |
| P15 |  |  |  | 0.504 ± 0.006a | 0.504 ± 0.020a | nd |  |  |  |  |  |  |
| P7  (Apigenin 6-C-arabinoside-8-C-hexoside) |  |  |  |  |  |  | 0.599 ± 0.024a | 0.506 ± 0.031a | 0.045 ± 0.006c | 0.353 ± 0.005a | 0.254 ± 0.054b | 0.0426 ± 0.021c |
| P20  (Apigenin-6-C-arabinoside-8-C-hexoside isomer 1) |  |  |  |  |  |  | 0.152 ±0.004a | 0.121 ± 0.017b | 0.041 ± 0.003c | 0.121 ± 0.003a | 0.087 ± 0.012b | 0.0453 ± 0.015c |
| P21  (Apigenin-6-C-arabinoside-8-C-hexoside isomer 2) |  |  |  |  |  |  | 0.859 ± 0.015a | 0.741 ± 0.025b | 0.038 ± 0.001c | 0.538 ± 0.004a | 0.374 ± 0.0599b | 0.0563 ± 0.045c |
| P25 (Tectoridin) | 0.288 ± 0.006a | 0.174 ± 0.036b | 0.266 ± 0.011a |  |  |  |  |  |  |  |  |  |
| P26 | 0.103 ± 0.024a | 0.099 ± 0.026b | 0.024 ± 0.020c |  |  |  |  |  |  |  |  |  |
| P33 (Chrysoeriol-7-O-glucuronide) |  |  |  | 1.467 ± 0.012a | 1.196 ± 0.088b | 0.047 ±0.088c |  |  |  |  |  |  |
| P43 (Chrysoeriol) |  |  |  | 0.166 ± 0.032a | 0.072 ± 0.015b | 0.199 ±0.019a | 0.048 ± 0.011 | 0.033 ± 0 | trace |  |  |  |

Data are the means ± SD (n = 3). Different alphabets in each row indicates a significant difference in phenolic content with a cereal variety. nd: not detected; trace: compound below limit of quantification; GAE: Gallic acid equivalent.

1. **PRx**

P14

P25

P26

P1

Basolateral side

Apical side

1. **PBx**

P2

P33

P43

P14

Basolateral side

Apical side

1. **PWx**

P21

P20

P17

P2

P2

Basolateral side

Apical side

1. **BWx**

P20

P17

P21

P2

Basolateral side

Apical side

**Figure S5.** Caco-2 cellular transport of polyphenols from digested pigmented grain extracts (PRx, PBx, PWx, BWx). Orange line: profile of phenolics in basolateral chamber; Green line: profile of phenolics in apical chamber. . PRx- Purple rice extract, PBx, purple barley extract, PWx – purple wheat extract, BWx- blue wheat extract


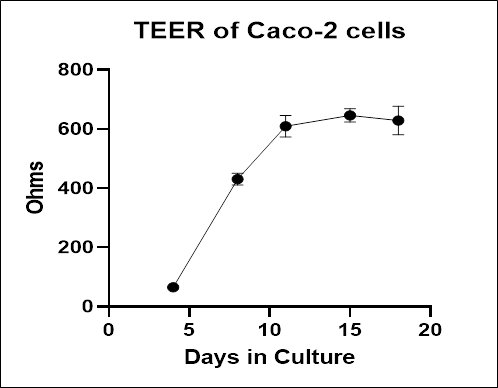


**Figure S6.** Formation of Caco-2 monolayer using trans-epithelial electrical resistance (TEER)

**P4**

**P15**

**P11**

**P19**

**P26**

**P39**

**P5**

**P6**

**P37**

**P38**

**P9**

**Figure S7**: Mass spectra of unidentified compounds from experiment
